# Development of Glycerol-Rose Bengal-Polidocanol (GRP) foam for enhanced sclerosis of a cyst for cystic diseases

**DOI:** 10.1371/journal.pone.0244635

**Published:** 2021-01-05

**Authors:** Soohyun Jeong, Sujin Kim, Youngjoo Choi, Han Na Jung, Kangwon Lee, Min Hee Park

**Affiliations:** 1 Program in Nanoscience and Technology, Graduate School of Convergence Science and Technology, Seoul National University, Seoul, Republic of Korea; 2 Program in Department of Applied Bioengineering, Graduate School of Convergence Science and Technology, Seoul National University, Seoul, Republic of Korea; 3 Center for Convergence Bioceramic Materials, Korea Institute of Ceramic Engineering and Technology, Cheo-ngju, Republic of Korea; Universita degli Studi di Bari Aldo Moro, ITALY

## Abstract

Polycystic kidney disease (PKD) is a common genetic disorder that results in a proliferating and enlarging cyst and ultimately leads to loss of kidney function. Because an enlarged cyst is a primary factor for limited kidney function, the large cyst is surgically removed by laparoscopic deroofing or sclerosant. This a relatively nascent treatment method entails complications and sometimes fail due to the cyst fluid refilling and infection. This study proposes using a more stable and effective polidocanol foam with glycerol and Rose Bengal (GRP form) to prevent cyst regeneration and irritation, which is caused by the required body movement during the treatment. Specifically, the foam retention time and viscosity were increased by adding glycerol up to 10% (w/v). The GRP form inhibited cellular proliferation and disrupted cellular junctions, e-cadherin, and cyst formation, demonstrated by the LDH, Live and Dead, and re-plating culture assays. The GRP foam was shown to be a safe and effective treatment as a commercial grade polidocanol foam form by an *in vivo* study in which subcutaneously injected mice injected with commercial 3% polidocanol, and the GRP foam showed no difference in inflammation. Thus, this study provides an advanced polidocanol form by adding glycerol and Rose-Bengal to help existing sclerotherapy.

## Introduction

Polycystic kidney disease (PKD) is a systematic disorder, and patients suffer from innumerable cyst proliferation and enlargement from the age of thirty. Multiple enlarged cysts lower kidney function and dramatically undermine patients' quality of life through flanking abdominal pain, infected cyst, or hypertension [1]. Therefore, relieving the cystic burden is essential in improving the clinical progression and safeguarding the quality of life of patients.

One method to ablate symptomatic renal cysts is sclerotherapy. It refers to introducing of a foreign substance into a vessel because it was most commonly used in scelrotherpay, or the site of injection to create cellular lining damage leading to the occlusion of the area. The lining cells that are exposed to the sclerosing solution exhibit activated calcium signaling and nitric oxide pathways, leading to cell death [2]. Sclerotherapy has been studied for renal cyst ablation for three decades [3], yet it is used in a case by case basis because sclerotherapy is only used for aberrantly large cysts [4]. Originally, doctors used an ethanol solution to ablate the cyst, which caused too much pain in the patients [3, 5]. Presently, active agents, such as polidocanol, are used for sclerotherapy. Studies have reported that polidocanol is safer and more effective than other sclerosants such as sodium tetradecyl sulfate (STS) [5–7]. Furthermore, the polidocanol product Asclera® is used in clinics for varicose veins, and 3% polidocanol has been used to ablate problematic cysts [8, 9]. The low content of polidocanol was especially developed for foam sclerotherapy. It is a more efficient and safer method than solution sclerotherapy because foam retains its form at the injection site and is more stable than solution [10]. Additionally, foam can effectively displace the surrounding liquid of the injection site and ensure increased contact time to fully contact with the tissue between the sclerosing agent and the targeted cells [11, 12]. Renal cysts and hepatic cysts are often ablated through foam sclerotherapy [5, 7–9, 13, 14]. Because thorough contact with the rostral part of the cyst is vital in preventing the regeneration of cysts, a longer foam retention time and shorter foam degeneration time are also important. To elongate the foam retention time, previous studies used thickening agents such as glycerol, xanthan gum, or xylene [15, 16]. Other than additives, previous studies also used various modalities to enhance foam stability [17, 18]. However, these studies did not demonstrate what effects these additives in the foam had on the sclerosing effect on the targeted cells and the possible adverse immunogenicity of the foam materials.

The utmost goal of the research is to investigate readily applicable and enhanced treatment methods for PKD patients. Thus, we selected 3% polidocanol foam as the base material. To induce a better retention time of the foam form, glycerol was also added in this study. Glycerol is a Federal Drug Administration (FDA) approved material used in clinics for other treatment methods. Bysimply mixing with polidocanol foam, it was speculated that the proposed material could be readily used for clinical studies [19]. Moreover, chromated glycerol or glycerol is also used in sclerotherapy; thus, we added it also to enhance the sclerotherapy effect [16]. In addition, Rose Bengal was added, which can induce collagen crosslinking and decrease infection possibility during treatment [20–22].

This study aimed to investigate these foam stabilizers' sclerosing effect and confirm their safe applicability *in vivo*. This study developed an advanced polidocanol form as a sclerosant by adding glycerol and Rose Bengal (RB) as a foam stabilizer [15, 16] and photosensitizer, respectively. Glycerol could augment the sclerosing effect and elongate the foam retention time [15]. Rose Bengal could also stabilize the foam and confer an additional effect by emitting ROS (Reactive Oxygen Species) after activation with a laser [23–27]. In addition, we examined the decreased cyst forming activity of Madin-Darby Canine Kidney (MDCK) cells after the foam treatment and clinical usability of this glycerol-Rose Bengal-polidocanol (GRP) foam by *in vivo* studies.

Currently, the sclerotherapy clinical scene has the following limitations. First, the patient needs to move to ensure the full coverage of the sclerosing solution. Second, cyst refilling occurs from an incomprehensive covering of the solution. Moreover, from the perspective of PKD treatment, Sclerotherapy was originally designed to treat of varicose veins, and thus, treatment for cyst ablation should be empirically studied. We hope that these studies will overcome the limitations occurring in the current clinical field of Sclerotherapy and develop a Sclerotherapy foam that is more suitable for PKD, which will be further utilized in the treatment of PKD diseases.

## Material and methods

### Foam preparation and analysis

The physical property of the foam was analyzed as previously described [15, 16] Foam viscosity was measured with the Brookfield DV2T Viscometer [28] An image of the foam was captured at each time point (0, 15, 30, 45, 60 seconds) up to 1 minute in RT. All foam was prepared by Tessari method with a solution to air ratio of 1:4.

### Cell culture and analysis

Madin-Darby Canine Kidney (MDCK) cells were purchased from ATCC (American Type Culture Collection, USA) and cultured following their instructions. The MDCK cells were cultured in a 3D collagen matrix, as previously described [29, 30] Briefly, Glutamine (200 mM, Thermo Fisher, USA), DMEM (Biowest, Korea), Hepes (Thermo Fisher, USA), and NaHCO_3_ (Sigma, USA) in 2 mg/ml collagen type 1 solution were mixed with MDCK cells up to 7 days.

For the foam-treated cellular LDH assays (LDH assay kit, Abcam, UK), cells were incubated at the bottom of the transwell (transwell plate, SPL, Korea) membrane and inserted by placing the insert upside down on a 6 well plate (Thermo Fisher, USA). MDCK culture medium was replaced with fresh media with no media on the top layer, and foam was added. The foam was removed by suction, and LDH activity was analyzed. Cultured MDCK cells were placed below the transwell membrane. The foam was added to the top layer for the direct interaction of the cells and foam. The foam was removed, and analysis followed. Next, 28 μl of the culture medium was placed onto the insert to protect the cells from drying out. Then, the cells on the transwell were placed in a 24 well plate and cultured for a day before the assay. When applying the foam to the transwell, the medium was removed from the inner insert. Then, 100 ul of foam was injected for 1 minute into each well. The media was collected immediately for the LDH assay. The LDH assays were done following the manufacturer's instructions. Live and dead (L/D) assays and cellular junction protein expression analysis were conducted by Image J.

### In vitro Rose Bengal (RB) analysis

Collagen (2 mg/ml, Thermo Fisher, USA) was prepared by mixing either PBS (Biowest, Korea) or PBS with different RB (Sigma Aldrich, USA) concentrations. We measured the absorbance at 548 nm or 315 nm (Fibrillogenesis) with a microplate reader.

### Animal studies

Eight-week-old male Balb/c mice (Orient bio, Korea) were grown under SPF (Specific-pathogen-free) conditions and allowed free access to food and water and maintained in a 12/12-hour light/dark cycle. In this study, animals were randomly divided into two separate groups of five mice per cage total of ten mice, treated into ethanol, and foam multiphoton laser (Leica, TCS SP8 MP, Germany) treatment group injected 1ml on dorsal subcutaneous of mice. The immune response evaluation of materials is generally administered in the abdominal or on the dorsal subcutaneous [31]. All animals to which samples were injected were included if an inflammatory response did or did not occur. However, if the animal died, the animal was excluded. It prevents early the collection of behavioral and histological data. The ethanol only treated group is control group comparing than to compare ethanol treated group and foam multiphoton laser treatment group, the biopsied subcutaneous mouse tissue samples were stained with hematoxylin-eosin for analysis of deep tissue treatment methods. The tissue slide was deparaffinized by incubating it at 60°C for one hour to melt the paraffin and was soaked in xylene three times for five minutes each. Deparaffinized slides were immersed in 100%, 95%, 80%, 70%, and 50% alcohol for five minutes in sequence to prepare them for staining. For H&E staining, after slide rehydration, the slides were stained with a hematoxylin-eosin working solution (Abcam, United Kingdom). Cell counts per unit tissue area of H&E images of each samples were analyzed by image J.

### Ethics statement

All animal experiments conformed to the National Health Guide for the Care and Use of Laboratory Animals and were approved by the Institutional Animal Care and Use Committee at Seoul National University (IACUC2018-2-15). Balb/c mice are commercially produced by Orient Bio. (Korea). The animal experiments in this study were approved by the experimental animal ethics committee of Seoul National University.

### Statistical analysis

Statistical significance was determined using the one-way analysis of variance (ANOVA) test. P < 0.05 indicated statistical significance. The data are presented as the mean and standard deviation for each condition. The number of repeats for each experiment was five.

## Results and discussion

Various percentages of polidocanol have been used in patients. However, 3% was selected in this study, which is the highest administered dosage possible and exerts the most cytotoxic and membrane destabilizing effect on MDCK cells, thus producing more comparable results. All foams were prepared using the standard Tessari method with a foam solution and air ratio of 1:4. The Tessari method was selected because it is one of the most often used methods to foam detergent sclerosants [16, 32, 33]. However, it must be noted there are other modalities to prepare sclerosants, as in a previous study by Xu et al [34].

**Fig 1:**
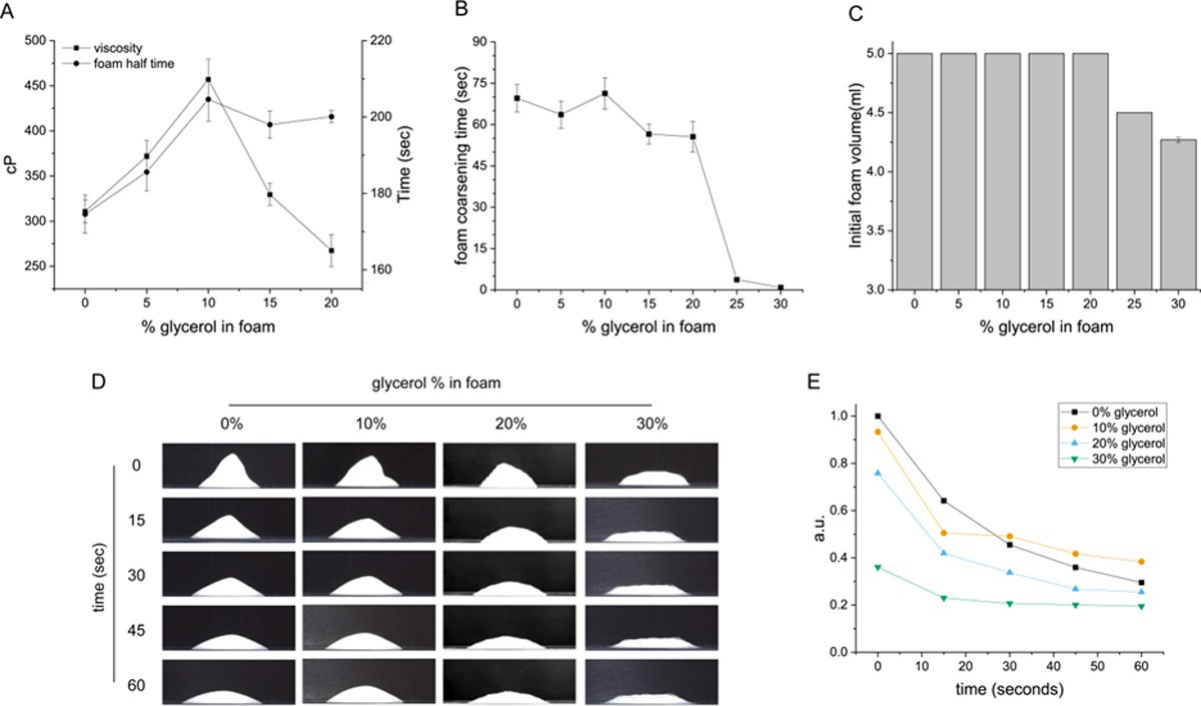
Glycerol enhances the sustainability of the polidocanol foam for cyst sclerotherapy. (A) Foam half time increases as glycerol is added up to 10% (v/v) and then decreases upon additional glycerol addition. The viscosity of the foam also decreased likewise. (B) Foam coarsening time. Foam coarsening time refers to the time when initial foam breakage occurred. (C) Initial foam volume. (D) Representative video capture image of foam. (E) Unregenerated foam height/width data for 60 seconds.

Glycerol was used as a potent factor for stabilizing the sclerosant foam in the polidocanol form. Glycerol in 3% polidocanol foam was examined as a function of the concentration of glycerol. 10% glycerol in polidocanol foam resulted in the most stable foam form at RT (room temperature) by viscosity (Fig 1A), foam coarsening time (Fig 1B), and initial foam volume (Fig 1C) [35, 36]. Fig 1D shows an image of the foam form, and Fig 1E shows the data for the ratio of the foam height to the width for how high the initial foam form was retained over time. The prolonged retention time of the foam form is important because it enables sclerosing of the rostral cystic lining cells without the patient moving during the treatment. The result for which the addition of 10% glycerol improves the foam stability is also comparable to a previous study [37], However, the relationship between the increased foam stability or the added glycerol amount to the foam efficacy or cytotoxicity has not been investigated for kidney cysts. There are reports in which various foams were tested *ex vivo*, in the venous tissue of patients [38]. However, to investigate *in vitro* at the cellular level the efficacy of the glycerol and Rose Bengal added GRP foam, a transwell experiment was devised shown later in result 3.

**Fig 2:**
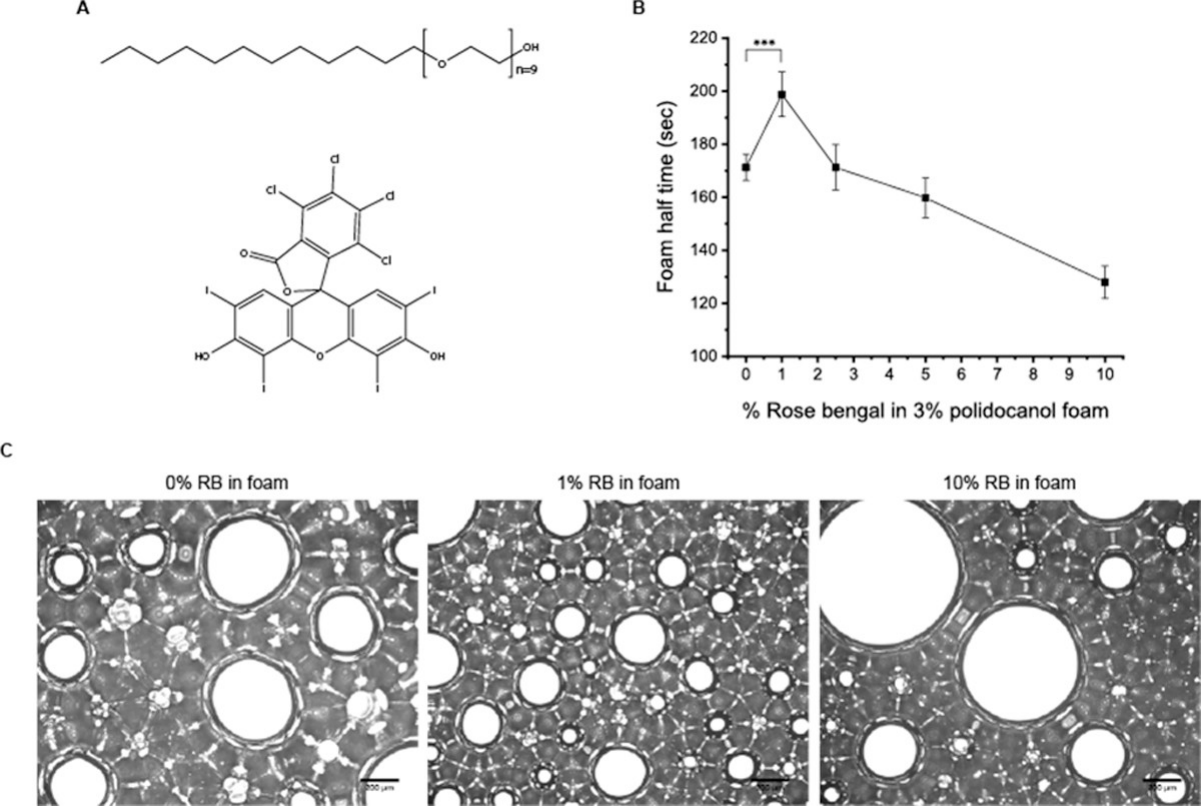
Structure of polidocanol and Rose Bengal and its effect on foam stability. (A) Structure of polidocanol (up) and Rose Bengal (below). (B) Foam half-time refers to the time until the foam degenerates into half of its original volume of solution. The 1% Rose Bengal content increased the foam stability by about 20%. (C) Microscopic image of the foam with various Rose Bengal concentrations. Bubble size and lamina thickness explain the increased and decreased foam half-time for the 1% and 10% Rose Bengal foam, respectively (scale bar = 200 μm).

RB (Fig 2A) was added to the polidocanol form to investigate multiphoton-activated collagen crosslinking of the organ cyst lining. Half time (Fig 2B) by various concentrations of RB showed that 1% (w/v) RB enhances foam stability, which is a novel finding because RB has not been tested for foam stability in previous studies. Microscopic imaging of the foam also demonstrates this point (Fig 2C). When using 1% RB foam at the same time point (– 10 s) after foam injection, the large held air bubbles were smaller in size than those of the 0% and 10% RB foam. Thus, these results suggest that RB also stabilizes the foam. The relationship between bubble diameter and foam stability can be expressed as a graph showing the Critical Coalescence Concentration (CCC) [39–42]. The critical coalescence concentration is the minimum frother concentration that effectively prevents air bubbles from joining during flotation in a flotation chamber. It is generally observed that the higher the molar mass of a frother, the lower its CCC value [43]. The size of the bubble is related to the CCC of the foam, and the CCC is shown to be related to the stability of the foam. Thus, in this study, it was shown that the foam containing 1% RB formed the most appropriate size bubble.

The effects of glycerol in the polidocanol form were investigated *in vitro*. Unfortunately, the experimental means for testing the effect of the foam on a cellular level are few. Some have reported gluing cells onto a plate before the foam treatment [44]. However, this would fail to mimic the real cystic environment. Many have reported *ex vivo* experiments using the veins of patients that are removed from their bodies and treated with foam [38]. However, because this study wanted to investigate the sclerosing foams on PKD organ derived cysts, ex vivo experiments using the veins were not deemed a reasonable tool in this study.

**Fig 3:**
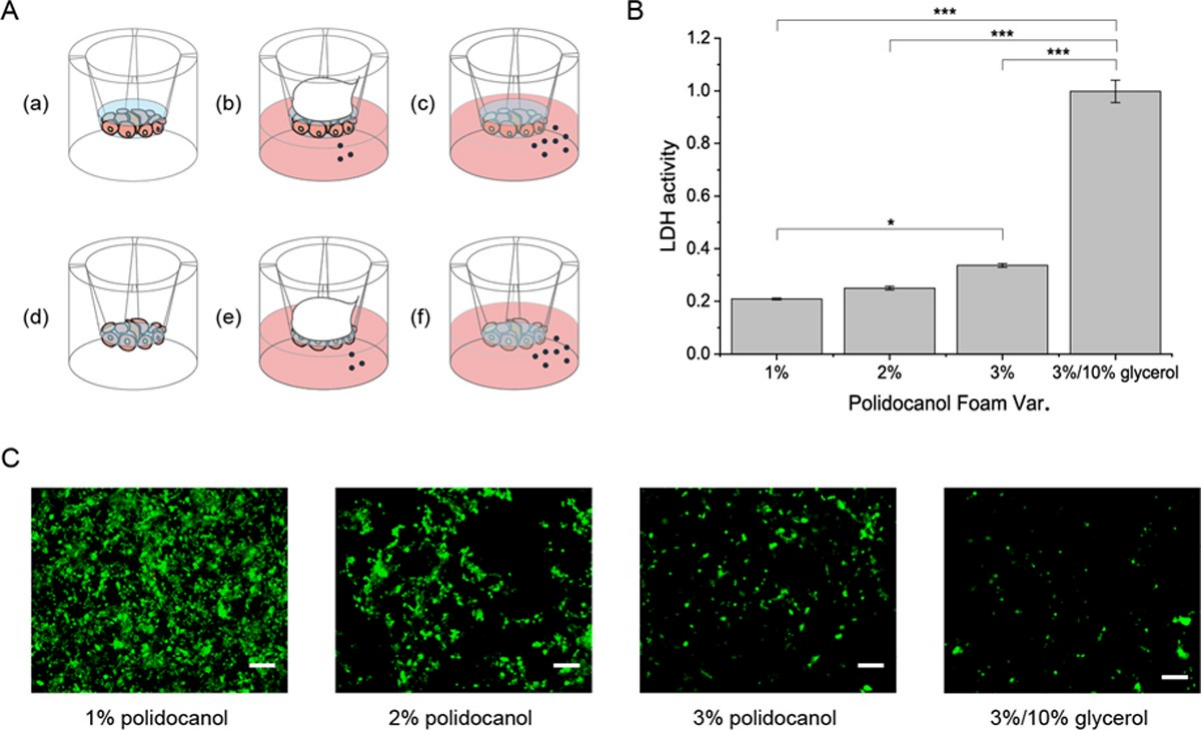
Glycerol in polidocanol foam better destabilizes cell membrane. (A) Scheme for *in vitro* foam analysis. (B) LDH results. (C) Live and dead image. No red signal is detected from all the cells affected by the foam, which are detached from the transwell membrane. (Scale bar = 100μm).

Hence, this study devised a quick and easy tool with the transwell system. Transwell is used to test cell migration and permeability. The membrane of the transwell is permeable for the foam to be in contact with the cells attached below and enables the handy removal of the foam with a pipet to retrieve the cells attached to the membrane. This study tested both 3D culture and 2D culture systems on the transwell (Fig 3A). The 3D culture system was the cyst forming activity of MDCK cells after the foam treatment, whereas the 2D culture system examined lactate dehydrogenase (LDH) activity or the degree of the destabilized cellular membrane caused by the foam. The results of the LDH assay showed that the more stable glycerol-polidocanol foam was more effective in cellular membrane destabilization (Fig 3B) and thus was more cytotoxic. This was also demonstrated by live and dead imaging of the transwell membrane, in which alive cells were still attached to the membrane and imaged as green. As known, increasing the concentration of polidocanol in the foam resulted in a decrease in live cells attached to the membrane (Fig 3C). The dead affected cells are considered to have fallen off. The inverse relationship between foam concentration and either the LDH activity or the number of green signals indicate that this study's method of testing the foam activity is a valid experiment method (Fig 3B and 3C). These results showed that the glycerol-polidocanol foam exerts an enhanced effect compared to any other foam tested in this study.

Another concern of the glycerol-polidocanol foam is whether it can cause extravesical necrosis when leaked onto other parts of the organ. Extravisional necrosis can bring extensive and even fatal organ failure and trauma [13]. Although foam sclerotherapy is less likely to cause extravesical necrosis than solution sclerotherapy, the cytotoxicity of the GRP form on MDCK cells was tested and compared with 3T3-L1 cells (S1 Fig). The LDH activity of 3T3-L1 cells from the glycerol-polidocanol foam was significantly lower than that of the MDCK cells. An increase of LDH activity shows that the concentration of polidocanol in the foam increases and bolsters the validity of this study's transwell foam testing method.

**Fig 4:**
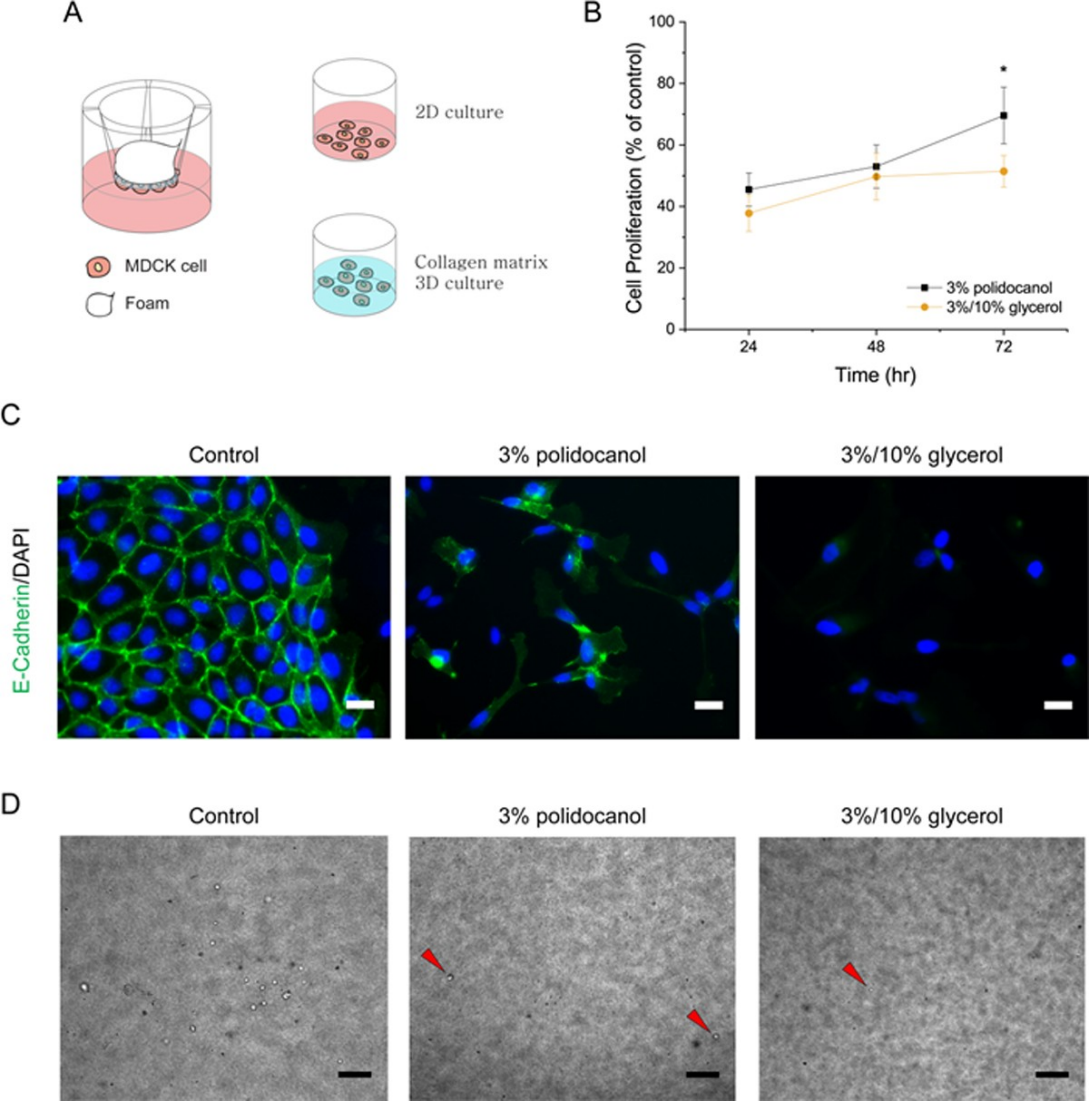
Glycerol in polidocanol foam attenuates proliferation and E-cadherin expression. (A) Scheme for *in vitro* foam analysis. After the foam treatment, the viable cells were collected and re-plated for proliferation and protein expression analysis. (B) The cell proliferation graph shows the augmented foam-treated cells proliferated slower compared to the polidocanol foam-treated cells. (C) Protein expression 3 days after cell re-plating. Augmented foam-treated cells failed to form a visible e-cadherin junction like the polidocanol-treated cells. (D) MDCK cells after 3D re-plating showed less cyst generation and a smaller cyst. (Scale bar = 200μm).

After confirming that the glycerol-polidocanol foam is more effective in destabilizing the MDCK cell membrane, the foam was investigated for altered cell proliferation and the expression of cellular junction protein by the cell re-plating method. Cells treated with the foam were washed, displaced from the transwell membrane, counted, and cultured either in a 2D without collagen matrix for proliferation and fluorescence imaging or in a 3D collagen matrix for cyst formation (Fig 4A). The results showed that the glycerol-polidocanol foam retarded cell proliferation more than the polidocanol foam alone. As shown in Fig 4B, the proliferation of MDCK cells after the polidocanol foam treatment increased, but the glycerol-polidocanol foam showed a lag phase from 48 to 72 hours. Moreover, the glycerol-polidocanol foam induced less E-cadherin protein expression, which was confirmed by immunofluorescence imaging (Fig 4C). The non-treated group was used as a control group. While both the control group and the polidocanol foam-treated group showed the expression of E-cadherin, the glycerol-polidocanol foam-treated group did not show explicit E-cadherin expression as much as the other two groups. The decreased or delayed expression of e-cadherin noticeable by the imaging suggest that the glycerol-polidocanol foam can better treat a PKD cyst or a cyst from various cystic diseases because e-cadherin is involved in the metastasis, cyst, and lumen generation, which are related to cyst refilling or regeneration [45–47]. Accordingly, fewer MDCK cells successfully generated a cyst after 7 days when the MDCK cells were treated with the foam and re-plated into a 3D collagen matrix. In comparison with the control group, the polidocanol foam-treated group and the glycerol-polidocanol foam-treated group both showed a significantly lower number of visible cysts (Fig 4D). However, the difference between the two different foam-treated groups was not significant, although the number of cysts from the glycerol-polidocanol foam-treated group was less than that of the polidocanol foam-treated group.

Herein, RB was not added to the foam because it could interfere with the imaging and the assay. However, we believe that the added RB would not significantly alter the result of this investigation because RB does not confer any additional cytotoxic effect (S2 Fig). Added RB with various concentrations in 3% polidocanol foam did not significantly alter the exerted LDH activity and cytotoxicity in the treated cells.

RB was used to enhance foam stability and elongate the time until foam degeneration for advanced sclerotherapy of the cyst (Fig 2B and 2C). In addition, the added RB as a component of the foam could enhance the effect. Hence, RB was used to ablate the cyst lining cells when activated by a laser. As shown in S3 Fig, this study did a test to confirm that RB can be delivered into the area by foam injection and a subsequent washing step as the standard sclerotherapy of a cyst. Collagen was placed on a 96 well plate, and the foam was injected with various concentrations of RB in the glycerol-polidocanol foam and then washed out. The well plate treated with the foam was red from the RB in the foam. When lighted with a 532 nm laser for 1 minute, the RB-treated collagen was more crosslinked in terms of rate (S3A Fig) and degree (S3B Fig). Additionally, this study showed that ROS was generated when treated with the RB-added foam and irradiated with a 532 nm laser for 1minute.

**Fig 5:**
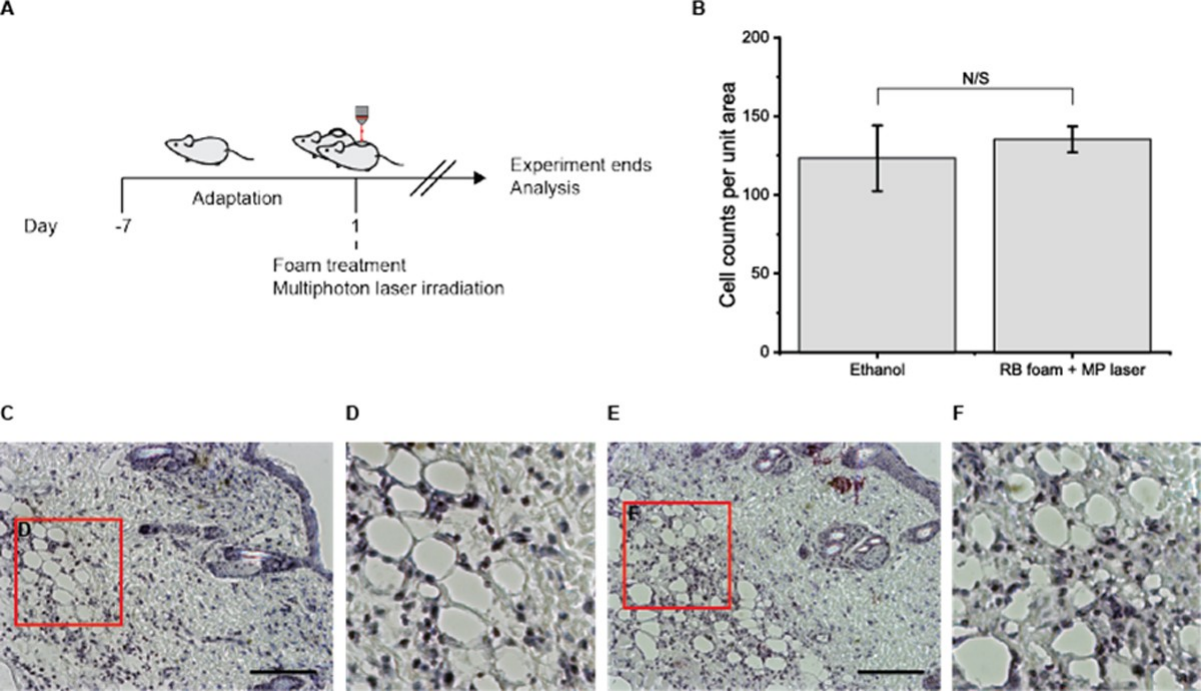
Multiphoton laser irradiation after rose-bengal-foam treatment suggests deep tissue treatment methods for cyst ablation. (A) schematic diagram of animal experiments. (B) Cell counts per unit area demonstrate that the immune cells on a given area of the image do not significantly differ from the ethanol and foam-multiphoton laser-treated group. (C-D) H/E representative image from the ethanol-treated group. (E-F) H/E representative image from the RB foam and the multiphoton laser-treated group (scale bar = 200 μm).

The safety and efficacy of the GRP foam were examined with an *in vivo* system and compared with a conventional sclerosant. Ethanol sclerotherapy is used conventionally to ablate a cyst in PKD patients. The GRP foam and ethanol were injected into the subcutaneous area of mice and irradiated with a multiphoton laser for RB activation (Fig 5A). After the treatment, the mice were sacrificed to investigate the acute immune response. H/E staining showed that the GRP foam was slightly more immunogenic than ethanol, but the result was not statistically significant (Fig 5B and 5C).

**Scheme 1:**
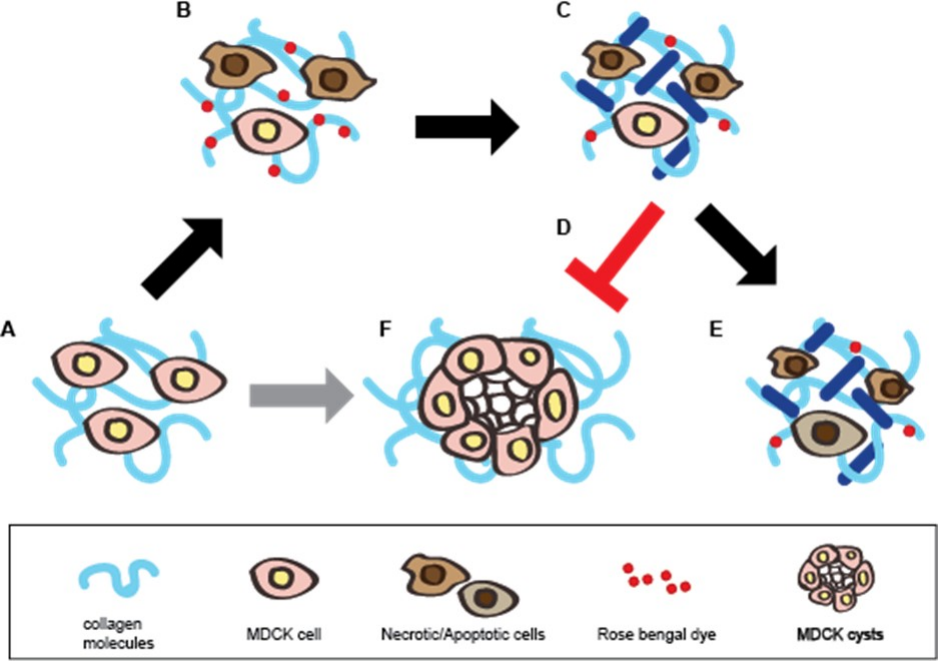
Schematic diagram of the mechanism of action of the glycerol-Rose Bengal-polidocanol foam. (A, F) MDCK cells proliferate and form a cyst (F) when cultured in a collagen matrix (A). (B) When treated with the Rose Bengal foam, polidocanol affects the cell membrane by ultimately killing the cells while Rose Bengal dyes the collagen matrix. (C) Green light activates the Rose Bengal and reactive oxygen species when Rose Bengal is emitted. (D, E) MDCK cells fail to proliferate and form a cyst (D) and fail to proliferate as the untreated cells (E). The dark blue line represents collagen crosslinking.

The overall scheme of the GRP foam is shown in Scheme 1. MDCK cells are known for their cyst forming activity inside the 2 mg/ml collagen matrix (Scheme 1F). However, ROS emission by RB and the effect of the GRP foam induce the MDCK cells to be less potent in cyst formation (Scheme 1D) and proliferation compared to the untreated cells (Scheme 1E) by treatment with the glycerol-RB-polidocanol foam (Scheme 1B) and laser (Scheme 1C) for RB activation within the 3D collagen matrix.

## Conclusions

In this study, an effective polidocanol foam was developed that not only retains its initial volume and height but also decreases the proliferation of the affected cells. MDCK, cyst forming cells that are used in PKD studies, was used to investigate the foam's ability to debilitate MDCK cyst generation by direct treatment and re-plating experiments. The induced GRP form destabilizes the cell membrane of MDCK and inhibits the expression of E-cadherin, a cellular junction protein that also has a vital role in cyst formation in PKD. In addition, the number of MDCK cells and the size of cysts were decreased by the GRP form in the 3D cell re-plating culture. Additionally, this study suggests a quick and handy way of testing sclerosing foams *in vitro* using a transwell system. Thus, this study proposed an advanced polidocanol form that includes glycerol and Rose-Bengal, which can facilitate PKD treatment research in sclerotherapy and provide a readily applicable means to better treat patients who suffer from endless pain and hardships.

## Supporting information

S1 Fig Policodanol-glycerol-foam is not as cytotoxic to T3TL1 cells as to MDCK cells. (DOCX)

S2 Fig Rose Bengal does not alter cytotoxicity of the polidcoanol-glycerol-rb-foam. (DOCX)

S3 Fig Rose Bengal delivered by foam is activated by 532nm laser irradiation and cause ROS generation. (Scale bar = 50μm).
(DOCX)
