# Supplementary figures and images for: Development of Glycerol-Rose Bengal-Polidocanol (GRP) foam for enhanced sclerosis of a cyst for cystic diseases
Source: PLoS One. 2021 Jan 5;16(1):e0244635. doi: 10.1371/journal.pone.0244635 (PMC7785218; doi:10.1371/journal.pone.0244635)

**
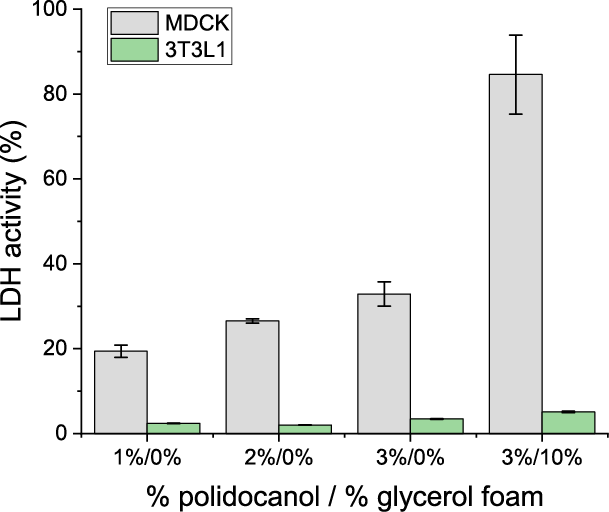
**

**S1 Figure. Policodanol-glycerol-foam is not as cytotoxic to T3TL1 cells as to MDCK cells**

Supplement: S1 Fig — (DOCX) [file pone.0244635.s001.docx]

**
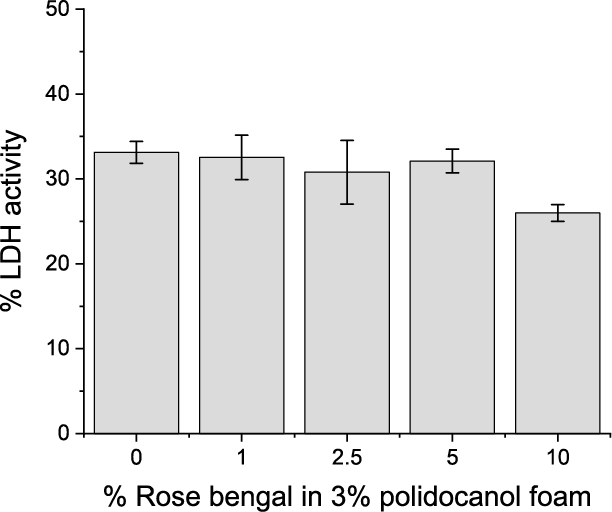
**

**S2 Figure. Rose Bengal does not alter cytotoxicity of the polidcoanol-glycerol-rb-foam**

Supplement: S2 Fig — (DOCX) [file pone.0244635.s002.docx]
