# Supplementary material for: Development of Glycerol-Rose Bengal-Polidocanol (GRP) foam for enhanced sclerosis of a cyst for cystic diseases
Source: PLoS One. 2021 Jan 5;16(1):e0244635. doi: 10.1371/journal.pone.0244635 (PMC7785218; doi:10.1371/journal.pone.0244635)
Supplement: S3 Fig — (Scale bar = 50μm). (DOCX) [file pone.0244635.s003.docx]

**
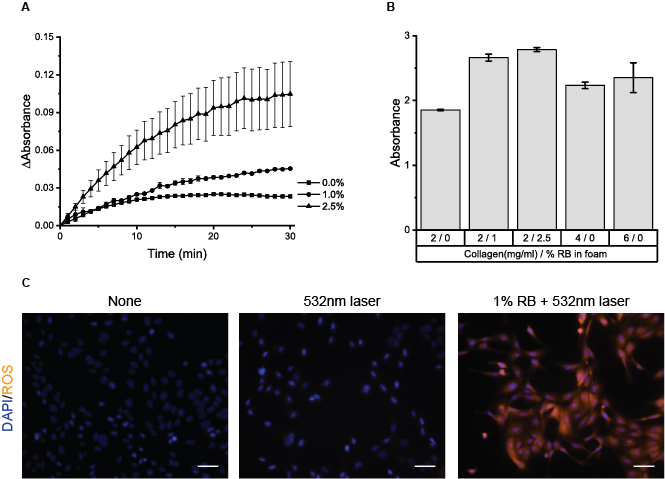
**

**S3 Figure. Rose Bengal delivered by foam is activated by 532nm laser irradiation and cause ROS generation. (Scale bar= 50μm)**
